# Are Cellulosome Scaffolding Protein CipC and CBM3-Containing Protein HycP, Involved in Adherence of *Clostridium cellulolyticum* to Cellulose?

**DOI:** 10.1371/journal.pone.0069360

**Published:** 2013-07-25

**Authors:** Pierre-Henri Ferdinand, Romain Borne, Valentine Trotter, Sandrine Pagès, Chantal Tardif, Henri-Pierre Fierobe, Stéphanie Perret

**Affiliations:** Aix-Marseille Université, CNRS, LCB UMR7283, Marseille, France; Universidad de Costa Rica, Costa Rica

## Abstract

*Clostridium cellulolyticum*, a mesophilic anaerobic bacterium, produces highly active enzymatic complexes called cellulosomes. This strain was already shown to bind to cellulose, however the molecular mechanism(s) involved is not known. In this context we focused on the gene named *hycP*, encoding a 250-kDa protein of unknown function, containing a Family-3 Carbohydrate Binding Module (CBM3) along with 23 hyaline repeat modules (HYR modules). In the microbial kingdom the gene *hycP* is only found in *C. cellulolyticum* and the very close strain recently sequenced *Clostridium sp* BNL1100. Its presence in *C. cellulolyticum* guided us to analyze its function and its putative role in adhesion of the cells to cellulose. The CBM3 of HycP was shown to bind to crystalline cellulose and was assigned to the CBM3b subfamily. No hydrolytic activity on cellulose was found with a mini-protein displaying representative domains of HycP. A *C. cellulolyticum* inactivated *hycP* mutant strain was constructed, and we found that HycP is neither involved in binding of the cells to cellulose nor that the protein has an obvious role in cell growth on cellulose. We also characterized the role of the cellulosome scaffolding protein CipC in adhesion of *C. cellulolyticum* to cellulose, since cellulosome scaffolding protein has been proposed to mediate binding of other cellulolytic bacteria to cellulose. A second mutant was constructed, where *cipC* was inactivated. We unexpectedly found that CipC is only partly involved in binding of *C. cellulolyticum* to cellulose. Other mechanisms for cellulose adhesion may therefore exist in *C. cellulolyticum*. In addition, no cellulosomal protuberances were observed at the cellular surface of *C. cellulolyticum*, what is in contrast to reports from several other cellulosomes producing strains. These findings may suggest that *C. cellulolyticum* has no dedicated molecular mechanism to aggregate the cellulosomes at the cellular surface.

## Introduction

Cellulose, a major polysaccharide on earth, is a linear polymer of glucose organized in a regular crystalline arrangement and forming insoluble linear microfibrils. In plant cell walls, these fibrils are surrounded by a complex matrix made up of other polysaccharides as hemicellulose or pectin [1], [2]. Several cellulolytic microorganisms carry out efficient deconstruction of crystalline cellulose and other polysaccharides of the plant cell wall. Among them, *Clostridium cellulolyticum*, a mesophilic anaerobic bacterium, produces highly active extracellular enzymatic complexes called cellulosomes together with free enzymes. In this cellulolytic strain, cellulosomes are made up of a non enzymatic scaffolding protein called CipC, composed of a CBM3, two hydrophilic modules (×2) whose function remains unknown and eight type I cohesins [3]. The cohesins bind with high affinity to the dockerin modules typically borne by the cellulosomal enzymes, thus leading to cellulosomes assembly [4]. Cellulosomal or free plant cell wall degrading enzymes display catalytic modules classified into three distinct groups in the CAZY database: the glycoside hydrolase, the pectate lyase, and the carbohydrate esterase group (http://www.cazy.org/ [5]).

Cellulolytic bacteria were early reported to bind to cellulose [6], [7], [8]. The adherence to their substrate is expected to bring them several competitive advantages: (i) the enzymes are secreted closer to the substrate, avoiding their diffusion in the extracellular medium, (ii) the hydrolysis products are released in the vicinity of the bacterium and can be directly consumed, thus limiting their diffusion and decreasing the feedback inhibition of the hydrolytic enzymes [8], [9]. Recently the cellulolytic bacterium *Clostridium thermocellum* was shown to form biofilm on cellulose [10], [11]. Cellulose was found to be significantly degraded in the biofilm area, compared to the areas without biofilm, highlighting the importance of cell adherence for cellulolytic activity.

In *C. thermocellum,* cellulosomes were shown to mediate cell binding to cellulose through the CBM3 borne by the cellulosomal scaffolding protein, CipA [6], [12], [13]. CipA contains a type II dockerin which interacts with type II cohesins hosted by 3 other non catalytic proteins OlpB, Orf2p, and SbdA [14], [15], [16]. These latter proteins are bound to cell surface through their Surface Layer Homology (SLH) modules. At the cell surface, cellulosomes form protuberances which can be observed using scanning electron microscopy [13]. These ultra-structures are missing at the surface of the non adherent *C. thermocellum* AD2 strain, which is no longer able to attach cellulosomes to the cell surface [6], [17]. In other cellulolytic species, such as *Clostridium cellulovorans*, *Acetivibrio cellulolyticus,* and *Bacteroides cellulosolvens*, similar ultrastructures were also observed [13], [18]. The cellulosomes were therefore hypothesized to be implicated in cellulose adherence process in these strains [12]. Molecular evidence supports this hypothesis: in the genome of *A. cellulolyticus*, and *B. cellulosolvens* genes encoding cell surface proteins were discovered which may mediate anchorage of the cellulosome scaffolding protein [12]. The anchorage would be done through type II cohesin/dockerin interactions as it was observed for *C. thermocellum*. *C. cellulovorans* lacks type II dockerin in the scaffolding protein CbpA. In this strain, cell binding to cellulose may be mediated by the cellulosomal enzyme Eng5E [19]. This protein may anchor cellulosomes to the cell surface thanks to the presence of a C-terminal type I dockerin and N-terminal Surface Layer Homology domains (SLH). In addition, hydrophilic modules of the scaffolding protein CbpA were shown to bind to *C. cellulovorans* cell wall fractions and were proposed to help to maintain the cellulosomes at the cell surface [20]. Thus in these species, the scaffolding protein of the cellulosomes seems to be directly or indirectly involved in cell adhesion to cellulose.

*Clostridium cellulolyticum* was shown to bind to cellulose [7], but in contrast to the cellulolytic species described above, the factors involved in this process have not yet been elucidated. In addition to the scaffolding protein CipC, the genome of *C. cellulolyticum* encodes 8 other putative CBM3-containing proteins [21]. Among them, seven were predicted to contain Family-9 glycoside hydrolase catalytic modules and are expected to be cellulases. They may be incorporated within cellulosomes since all of them bear a dockerin module. The eighth CBM3-containing putative protein is the product of the gene located at the locus Ccel_1491. Annotation of this gene in NCBI database indicates that the corresponding protein contains a CBM3a, similar to the CBM3 of CipC which is known to bind strongly to crystalline cellulose [22]. Moreover, it is a very large protein of 250 kDa, of unknown function, and for which computational analysis failed to predict any catalytic-, dockerin-, or cohesin-module [21]. The presence of such a protein in *C. cellulolyticum* prompted us to analyze its function and its putative role in cell adhesion to cellulose as well as that of the scaffolding protein CipC, since cellulosomal scaffolding proteins were proposed to mediate cell adhesion in several other cellulolytic bacteria.

## Results

### Bioinformatic analysis of the protein encoded by the gene at the locus Ccel_1491

**Figure 1:**
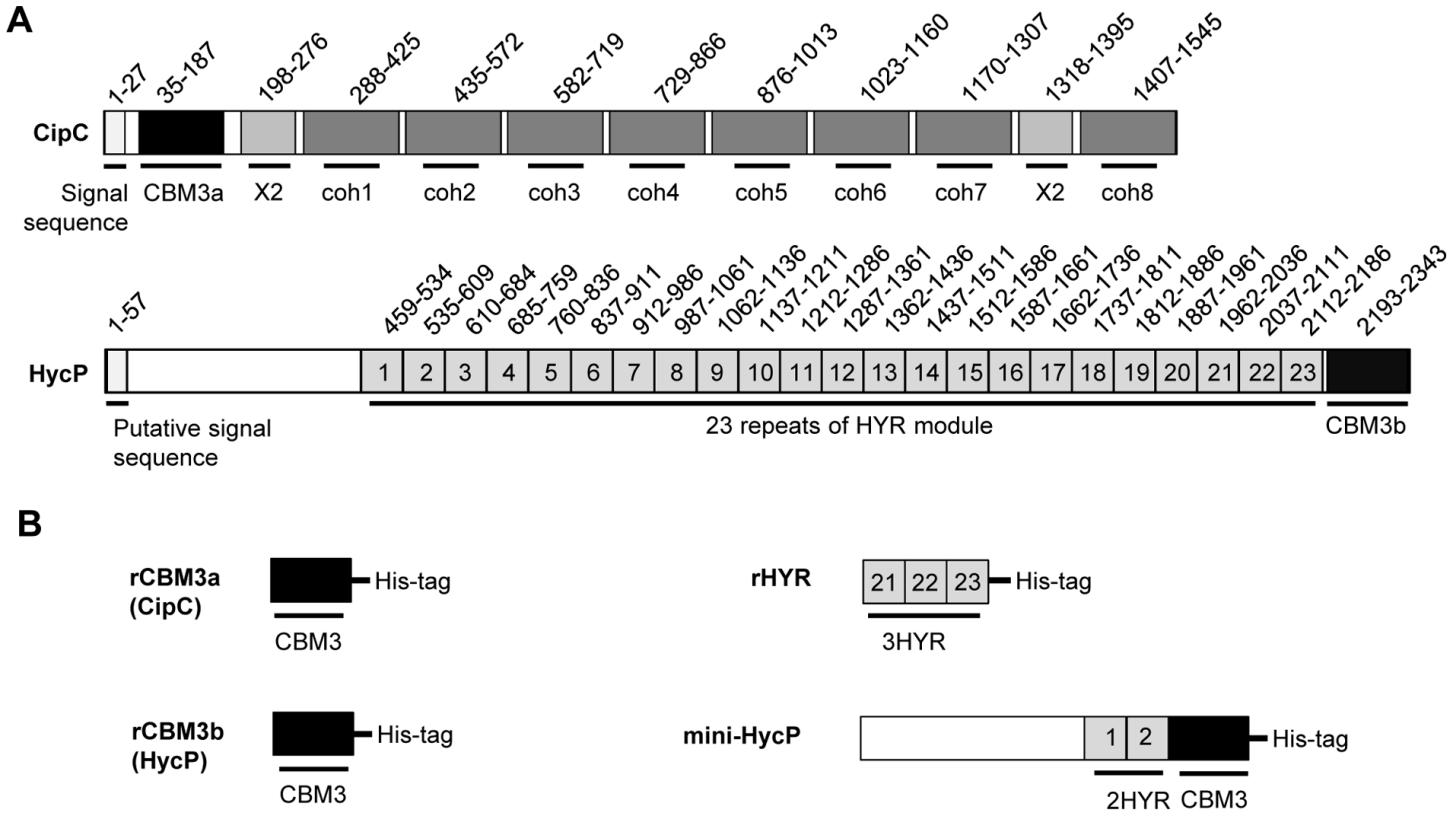
Structural organization of the proteins used in this study. A. Structural organization of CipC and HycP from *Clostridium cellulolyticum.* Numbers above the protein correspond to the starting and final amino acids of each module in the full length molecule. The numbers in HycP indicate the order of the HYR modules starting from the N-terminus. HYR modules were identified using PFAM, SMART and by manual search (see alignments in data S1). B. Modular organization of recombinant proteins produced in the present study. The numbers of the HYR modules in recombinant proteins correspond to the same HYR modules in the wild-type protein.

The structural organizations deduced from bioinformatic analysis of the product of the gene at the locus Ccel_1491 and of CipC are presented in figure 1. CipC is a well described protein of 160 kDa which contains eight cohesins, and a CBM3 at the N-terminus. It is a secreted protein and its precursor harbors a typical gram positive signal peptide. In contrast, the newly identified product of the gene present at the locus Ccel_1491 is predicted either as a secreted protein with a putative 58 amino acids long signal peptide or as a membrane protein with a transmembrane helix located between amino acids 34 and 51. This 250-kDa protein exhibits 23 copies of a hyaline repeat module (HYR) and a CBM3 at the C-terminus. For convenience, the product of the gene at locus Ccel_1491 will be named HycP for HYR modules and CBM3 containing protein. At the N-terminus of HycP, a region of about 250 amino-acids does not match with any other classified domain except with a bacterial Ig-like domain family 3 (BID_1 in SMART database), with a very weak E-value. This domain is usually found in bacterial cell surface proteins.

HycP is composed of 23 copies of HYR modules which account for nearly 75% of its sequence. Each HYR module is about 75 amino-acids long; an alignment of these modules is presented in data S1. The HYR modules were initially discovered in the hyalin protein found in the echinoderm extra-embryonic matrix and are responsible for the recognition of this protein by its cell surface receptor [23]. The hyalin protein contains exclusively this type of repeated modules. HYR modules belong to the immunoglobuline like fold, like the Fn3 domain [24]. They are found in eukaryotes as well as in prokaryotes where they are detected in some surface proteins, associated with Family-18 glycoside hydrolase modules (chitinase) or as part of hypothetical proteins. Their function in these proteins is unknown [24]. For HycP no obvious function can be deduced from a structural organization analysis neither did the genetic environment give further clues, since the gene encoding HycP is framed upstream and downstream by genes of unknown function.

### Classification and characterization of HycP CBM3

**Figure 2:**
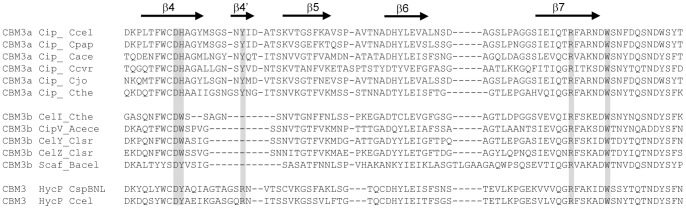
Amino-acid sequence alignment of CBM3a and CBM3b from various CBM3a or CBM3b containing proteins. Alignment has been performed using ClustalW2. It is focused on residues considered to participate in planar interaction with cellulose, highlighted in grey box. Regions of secondary structure are marked with an arrow and labeled as in the structure of the CBM3a of the scaffolding protein CipA from *Clostridium thermocellum* (Tormo 1996). CBM amino-acid sequence aligned (accession numbers codes in parentheses) are: Cip_Ccel(YP_002505087) and HycP_Ccel (YP_002505824) from *Clostridium cellulolyticum*; HycP_CspBNL (YP_005147316) from *Clostridium sp. BNL1100*; Cip_Cpap (ZP_08194681) from *Clostridium papyrosolvens*; Cip_Cace (NP_347546) from *Clostridium acetobutylicum*; Cip_Ccvr (ZP_07630535) from *Clostridium cellulovorans*; Cip_Cjo (BAA32429) from *Clostridium josui*; Cip_Cthe (ZP_14248391) and CelI_Cthe (AAA20892) from *Clostridium thermocellum*; CipV_Acece (AAF06064) from *Acetovibrio cellulolyticus*; CelY (YP_007373484) and CelZ (CAA39010) from *Clostridium stercorarium*; Scaf_Bacel (AAG01230) from *Bacteroides cellulosolvens*.

HycP contains a CBM3 found at the N-terminus of the protein. The CBM3 family is sub divided in several subfamilies. The CBM3 found in CipC belongs to the CBM3a subfamily as those in other scaffolding proteins [25], [26]. A sequence search in the NCBI data bank for microbial proteins sharing similarity with the CBM3 of HycP provided a list of proteins containing CBM3a or CBM3b. The two highest scores were obtained with the CBM3b of the exoglucanase CelY from *Clostridium stercorarium* (accession number gi|1708082) and the CBM3a of the scaffolding protein CipA from *C. thermocellum* (accession number gi|2554721). In order to further analyze the sequence of HycP CBM3, an alignment with several known CBM3a and 3b sequences was performed (figure 2). As it was formerly shown, the presence or absence of a short 4′ β-strand allows discrimination between CBM3a and CBM3b, respectively [26]. This strand holds a tyrosine which is one of the conserved amino-acids important for the binding of the CBM3a to the planar crystalline cellulose [27], [28], [29]. In contrast to the CBM3b, HycP CBM3 contains an additional stretch of about 8 amino-acids. This stretch lacks the conserved tyrosine found in the 4′ β-strand of the CBM3a, and does not contain any aromatic residues. Remarkably a conserved histidine found in all CBM3a in the 4 β-strand, is replaced by an aromatic amino acid in the HycP CBM3, as observed in the case of the CBM3b subfamily (fig 2). These observations lead us to propose the classification of the CBM3 from HycP in the CBM3b rather than in the CBM3a subfamily.

**Figure 3:**
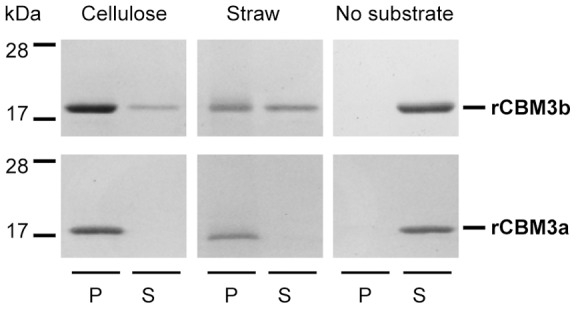
Interactions of rCBM3a and rCBM3b with straw and crystalline cellulose. Recombinant proteins were mixed with substrates during one hour. After centrifugation the bound proteins found in the pellet (P), and the unbound proteins present in the supernatant (S), were analyzed by SDS-PAGE.

Both CBM3a and 3b are known to bind to crystalline cellulose. We analyzed the cellulose binding capacity of the newly discovered CBM3b and compared it with that of the well known CBM3a from the scaffoldin CipC. Recombinant CBMs, referred to as rCBM3a and rCBM3b for respective proteins CipC and HycP (figure 1B), were fused to a polyhistidine tag at the C-terminus, produced in *Escherichia coli*, purified and used for binding assays. Binding capacities were first investigated on crystalline cellulose and straw. rCBM3a was shown to bind strongly to both crystalline cellulose and straw, whereas rCBM3b seems to have higher affinity for cellulose than for straw (figure 3). We measured the dissociation constant for both CBMs on different cellulosic substrates (Table 1). Both modules bind to phosphoric acid swollen cellulose (PASC) with the same affinity, but the rCBM3b displays approximately 10 times lower affinity on all tested crystalline cellulose (Sigmacell, Avicel and BMCC) compared to the rCBM3a. Nevertheless our results indicate that the CBM3b is functional and is able to bind to crystalline cellulose, but with lower *K_D_* values ranging from 10^−5^ to 10^−6^ M.

**Table 1:** Dissociation constants of rCBM3a and rCBM3b to cellulosic substrates.

	K_D_ (M)	
Substrate	rCBM3a (CipC)	rCBM3b (HycP)
BMCC	2.49E^−8^	9.5E^−6^
Avicel	8.017E^−7^	1.16E^−5^
Sigmacell	4.33E^−7^	8.83E^−6^
PAS-cellulose	8.51E^−7^	7.47E^−7^

### HycP enzymatic assays

The HycP protein contains 23 HYR modules along with a functional CBM3b. As CBM3b-containing proteins are often cellulases, we explored if HycP has a catalytic activity towards various cellulosic substrates. In order to facilitate these tests we produced a shortened form, called mini-HycP in *E. coli* which is composed of the first 409 amino-acids found after the predicted signal sequence cleavage site (the part of the protein which does not match with any conserved domains), the two first HYR modules (which were not reported to display catalytic activity) and the CBM3b (figure 1B). Mini-HycP also contains a C-terminal His-tag to facilitate its purification. Activity assays were performed on straw, crystalline cellulose (Avicel), phosphoric acid swollen cellulose, and Carboxy-Methyl Cellulose (CMC) by measuring the quantity of reducing sugars released. Under our experimental conditions (37°C, pH 6), we were not able to detect any activity of the mini-HycP on any of these substrates (data not shown).

### Construction of *hycP* and *cipC C. cellulolyticum* mutant strains

Our results showed that HycP has a functional CBM3b but no enzymatic activity toward cellulosic substrates. We therefore explored the possibility that the function of HycP is to induce the binding of *C. cellulolyticum* to cellulose, since the protein contains a cellulose binding module, as well as numerous HYR modules which are found in many cell surface proteins [24]. Furthermore bioinformatic analyses of the sequence predicted a putative transmembrane helix at the N-terminus. In order to verify this hypothesis, we constructed a mutant strain from *C. cellulolyticum* in which the *hycP* gene was inactivated using the ClosTron technique developed by Heap and co-workers [30]. As scaffolding proteins are reported to be involved in cell adhesion to cellulose in several cellulosome-producing bacteria, we decided to inactivate the *cipC* gene as well, using the same technique. The intron was designed to target the very beginning of *cipC* in the DNA region encoding the CBM3a module, in order to prevent any production of a truncated CipC form of the protein that would still display the cellulose binding module (figure 1A). Two mutant strains were thus constructed, MTL*cipC* and MTL*hycP*.

**Figure 4:**
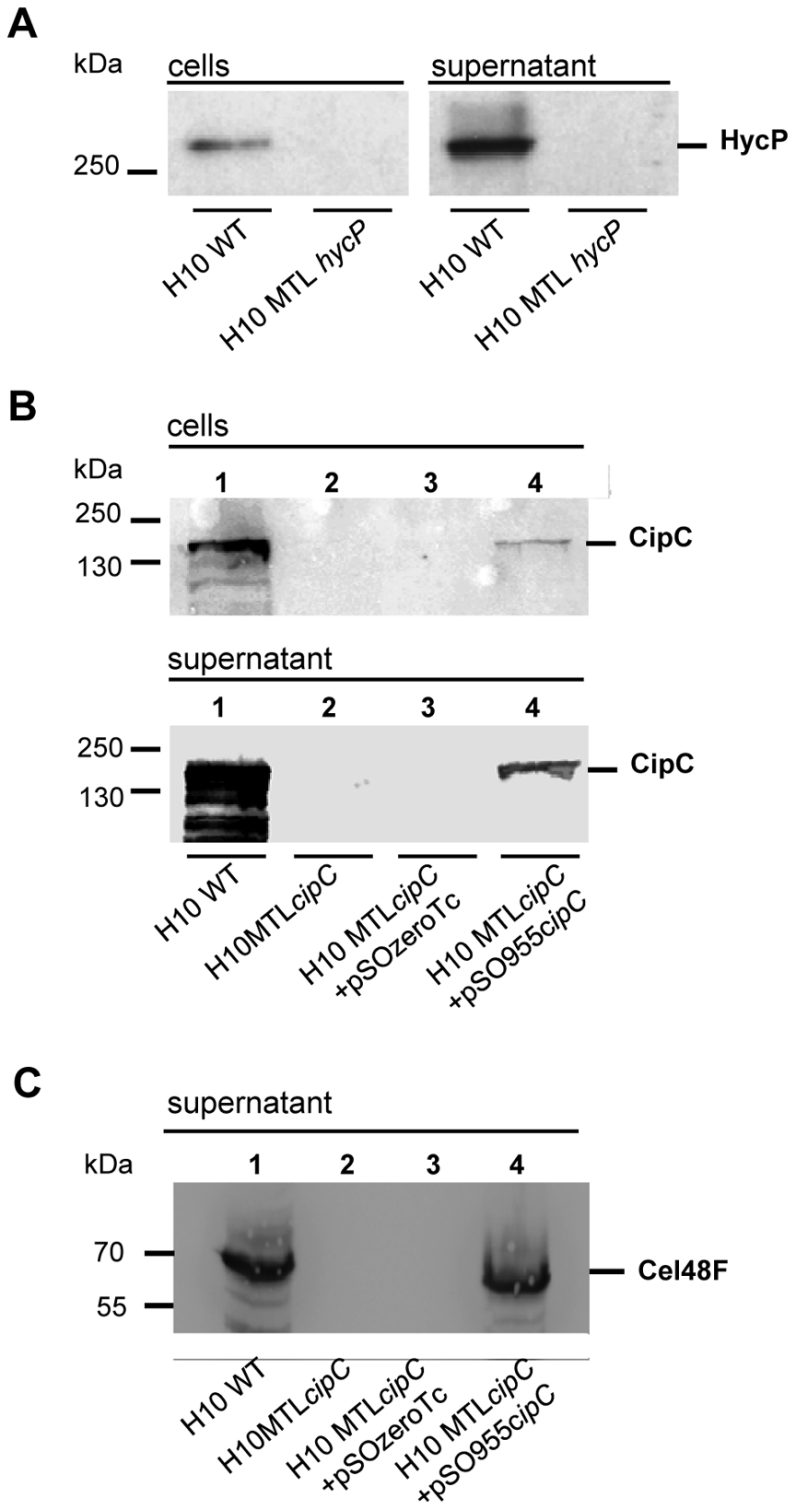
Detection of HycP CipC and Cel48F in different *Clostridium cellulolyticum* strains. Different *C. cellulolyticum* strains were studied: wild-type strain, MTL*hycP*, MTL*cipC*, MTL*cipC*(pSOS955*cipC*) and MTL*cipC*(pSOSzero-Tc) strains. Aliquot was taken from a culture of these strains at the exponential growth phase on cellobiose substrate, and centrifuged to separate the cells and the supernatant. Cell fraction and 10% TCA precipitated supernatant fraction corresponding to the same culture volume were subjected to SDS-PAGE. After transfer onto nitrocellulose membranes, membranes were probed with antibodies directed against HYR modules from HycP (Panel A), or CipC (Panel B), or Cel48F (Panel C).

Analysis of the genomic DNA of both strains by PCR and southern blot confirmed the genetic localization of the mutations and the presence of only one insertion in the chromosome (data not shown). In the strain MTL*cipC*, the pMTL007*cipC* vector was cured but not in the strain MTL*hycP* where the pMTL007*hycP* persisted in all the tested clones obtained from two transformation events, even after many replicates. In order to detect HycP in the different strains we used rabbit antibodies raised against rHyr, a purified recombinant protein produced in *E coli* and containing the three C-terminal HYR modules fused to a C-terminal His-tag (Fig 1B). Analysis of both, whole cells and cellobiose culture supernatant of each mutant, indicated that the proteins of interest were absent, while in wild-type cells bands corresponding to proteins of about 280 kDa and 160 kDa were detected using anti-HYR or anti-CipC CBM3a antisera, respectively (fig 4 A and B lanes 1 and 2). In addition, we observed that in the wild-type strain, HycP is more abundant in the supernatant than in the cell fraction, thus indicating that this protein is mainly secreted. The same observation was made concerning CipC what in this case is consistent with its typical gram positive signal sequence. The presence of proteins HycP and CipC in the cellular fraction of the wild-type strain may either be due to their production in the cell prior to secretion, or to a putative association with the cell wall.

### Cell binding to cellulose and growth analysis of the MTL*hycP* mutant strain

**Figure 5:**
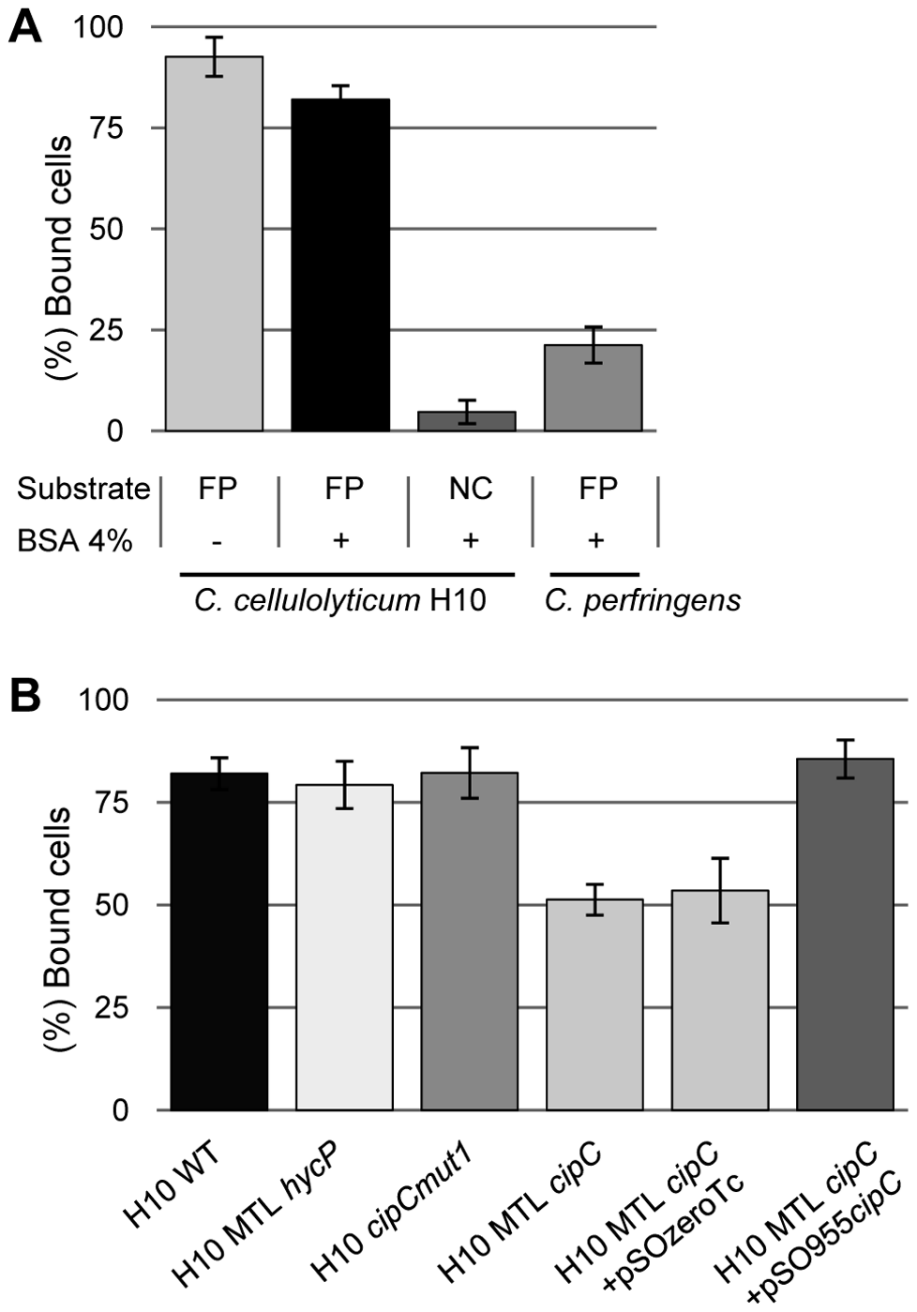
Cell adherence to cellulosic substrates. Cells cultured on cellobiose were incubated one hour in anaerobic conditions with a strip of insoluble substrate. Binding percentage is calculated from the level of unbound cells measured in the supernatant by spectrophotometry (optical density at 450 nm) compared to the optical density value of an assay where no insoluble substrate was added. (A) *Clostridium cellulolyticum* is incubated on filter paper (FP) or on nitrocellulose (NC) strips with or without BSA saturation and compared to the binding level of *Clostridium perfringens* to BSA saturated filter paper. (B) Cellulose binding capacity of *C. cellulolyticum* wild-type strain, MTL*hycP*, MTL*cipC*, MTL*cipC*(pSOS955*cipC*), MTL*cipC*(pSOSzero-Tc), and *cipCmut1* mutant strains. Experiments were performed in triplicates, on at least three independent experiments and two isolated clones for MTL*hycP*, MTL*cipC*, MTL*cipC*(pSOS955*cipC*) and MTL*cipC*(pSOSzero-Tc).

In order to test the MTL*hycP* mutant strain for its ability to bind to cellulose we used a spectrophotometric adhesion test. It indicated that 95% of wild-type *C. cellulolyticum* cells cultured in cellobiose, bind to filter paper cellulose (figure 5A). In presence of BSA, which reduces the unspecific binding, still 80% of *C. cellulolyticum* cells bind to cellulose whereas only 10% were found to bind to nitrocellulose, which is a chemically modified cellulose. For comparison *Clostridium perfringens*, a human pathogen unable to grow on cellulosic substrates, showed only 20 % of adherent cells on filter paper, thus confirming the specificity of the test. Subsequent adhesion tests were all performed in presence of BSA in order to study specific binding. Observations by scanning electron microscopy (SEM) of the filter paper after cell adhesion confirmed the presence of bound cells (data not shown).

**Figure 6:**
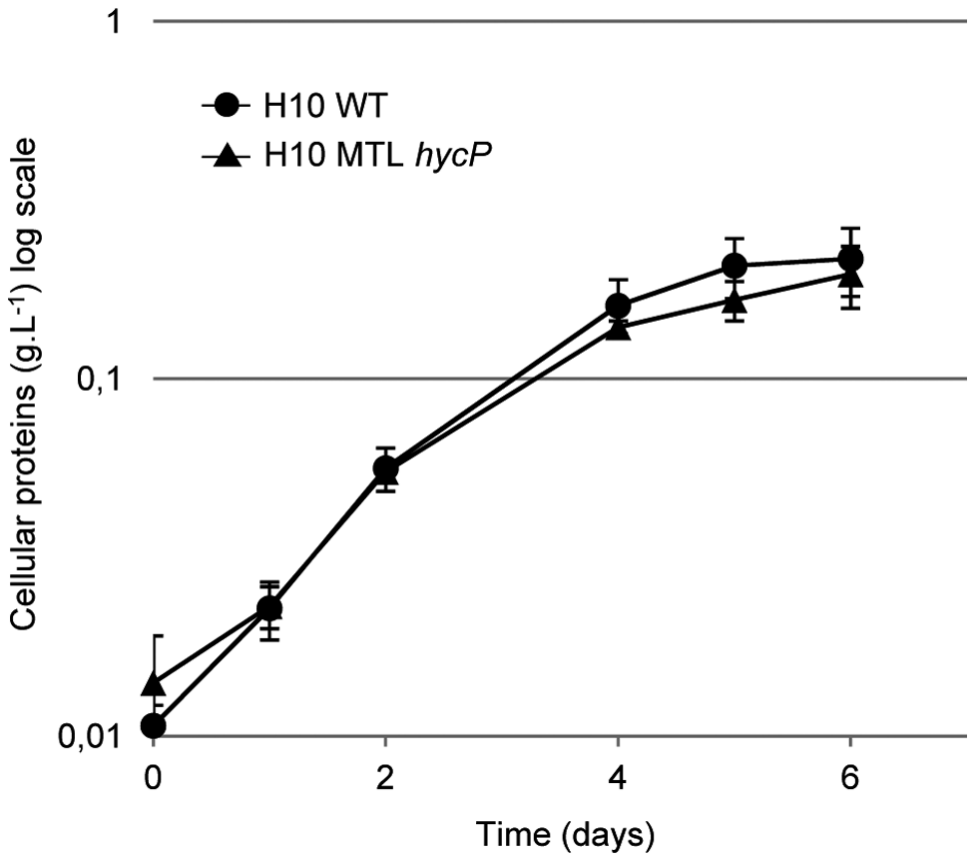
Growth of *Clostridium cellulolyticum* wild-type and MTL*hycP* strains on cellulose. Both strains were grown on rich medium containing 5 g.L^−1^ Sigmacell. Growth was monitored by measuring total protein content. Experiment was performed in duplicates. Growth performed in other condition as minimal medium containing Sigmacell or Avicel did not show any differences between both strains.

The MTL*hycP* mutant strain was assayed for its binding capacity to cellulose. The results indicated that MTL*hycP* mutant cells bind to cellulose at the same level as the wild-type strain, thereby demonstrating that HycP has no obvious role in cell binding to cellulose (fig 5B). To further analyze the role of HycP, we measured the growth of the MTL*hycP* mutant strain in various conditions and compared them with the wild-type strain. On cellobiose rich medium, generation time of MTL*hycP* mutant was 25% lower than that of wild-type strain, indicating that when HycP is not produced and not secreted, the fitness of *C. cellulolyticum* on soluble sugars is enhanced. When using insoluble cellulose as the substrate, no significant difference was observed whatever medium (rich or minimal medium) or crystalline cellulose (Avicel or Sigmacell) were used (fig 6).

### Cell binding to cellulose and analysis of the MTL*cipC* mutant strain

CipC is the first gene of an operon containing 12 genes which encode mainly glycosyl hydrolases (*cel48F*, *cel8C*, *cel9G*, *cel9E*, *orfX*, *cel9H*, *cel9J*, *man5K*, *cel9M*, *rgl11Y*, *cel5N*), directly involved in plant cell wall degradation [31], [32]. We used two different *cipC* mutant strains: the new MTL*cipC* mutant constructed in the present study, and a spontaneous mutant *cipCmut1* which was formerly characterized [31]. This mutant strain contains an insertion sequence at the 3′ extremity of the *cipC* gene leading to the production of a truncated CipC. The presence of the insertion sequence in the *cipC* gene induced a polar effect which caused the abolishment of the expression of all other genes localized in the operon downstream of *cipC* [31]. We analysed the binding capacities of both strains. We observed that *cipCmut1* binds to cellulose at the same level as the wild-type strain. As this strain produces none of the cellulases encoded by the operon *cip-cel,* our observation might indicate that these proteins do not participate in cell binding. In the MTL*cipC* mutant strains, only 50% of the cells bound to cellulose. We observed that in this strain, already the second gene downstream *cipC,* namely *cel48F,* was not expressed, suggesting the occurrence of the same polar effect as in the *cipCmut1* strain (fig 4C, lane 1 and 2). The difference between MTL*cipC* and *cipCmut1* strains is therefore that the MTL*cipC* mutant strain does not produce any CipC, whereas the *cipCmut1* mutant strain still produces a small amount of a truncated form of CipC with the N-terminal CBM3a [31]. This may explain their difference in cellulose adherence and suggests the involvement of CipC, through its CBM3a, in cell binding to cellulose.

In order to validate the involvement of CipC in the phenotype of the mutant constructed in the present study, we complemented MTL*cipC* strain using a replicative vector (pSOS955*cipC*). It allows the expression of the *cipC* gene under control of a constitutive promoter which has been shown to be functional in *C. cellulolyticum* [33], [34], [35]. A control strain MTL*cipC*(pSOSzero-Tc) containing the same vector but without the expression cassette, was also constructed. Both strains were analyzed for their CipC content by western blot, along with the wild-type strain. As expected, the complemented strain MTL*cipC*(pSOS955*cipC*) produced CipC. However the production of Cel48F was also detected, suggesting that a homologous recombination event occurred between the *cipC* copy present in the chromosome and the one of the vector, restoring the expression of the operon (fig 4B and C, lanes 3 and 4). Recombination events occurred in all clones obtained after two independent transformation events. Despite this observation, we measured the capacity of the complemented and the control strain to bind to cellulose. The MTL*cipC*(pSOS955*cipC*) complemented strain showed equal binding capacity as the wild-type strain, in contrast to the control strain MTL*cipC*(pSOSzero-Tc), indicating that the complementation restores the fully adherent phenotype (fig 5B). In summary these data strongly suggest that CipC participates in binding of *C. cellulolyticum* to cellulose while HycP does not.

### Observation of the cell surface

**Figure 7:**
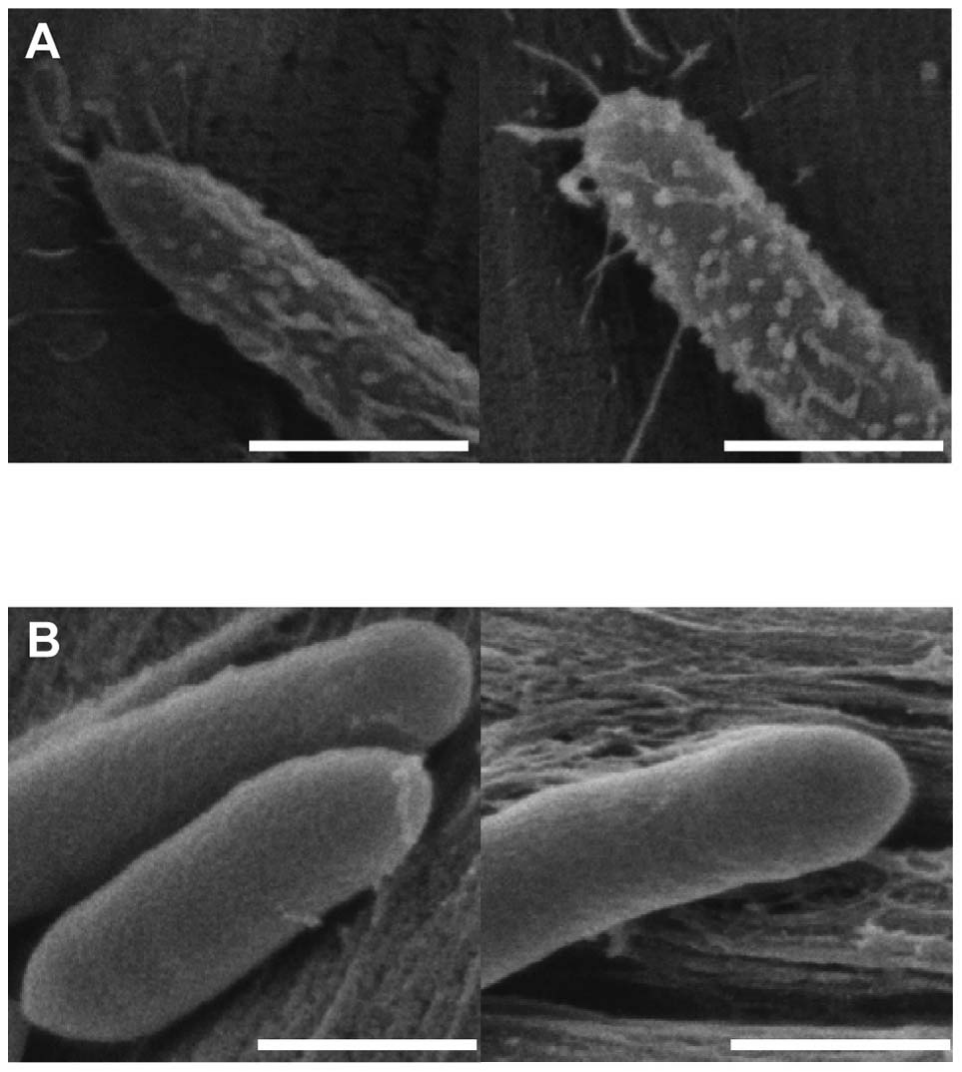
SEM observation of *Clostridium cellulolyticum* and *Clostridium thermocellum* grown on filter paper. *Clostridium thermocellum* (A) and *Clostridium cellulolyticum* (B) were grown on filter paper. Pictures are representative of two independent experiments. Bar represents 500 nm.

Some cellulolytic bacteria are able to form cellulosomal protuberances at their surface which participate in cell adhesion on cellulose and are composed of cellulosomes. The presence of these protuberances has never been shown for *C. cellulolyticum*. As shown above, in *C. cellulolyticum* CipC is involved in its adhesion to cellulose. To detect if on the surface of *C. cellulolyticum* also protuberances are formed, we observed the cell surface of *C. cellulolyticum* during the growth on filter paper using SEM and compared it to the surface of *C. thermocellum* grown on the same substrate. Wild-type *C. cellulolyticum* cell surfaces were entirely smooth and lacked ultra-structural protuberances, in contrast to *C. thermocellum* whose cell surfaces displayed many protuberances (fig 7).

## Discussion

The CBM3 family contains several subtypes, among them the CBM3a and the CBM3b are known to bind strongly to crystalline cellulose [22], [26], [27], [36]. In the present study, we analyzed the properties of the new HycP CBM3 and compared them to those of the well known CipC CBM3a (Table 2). We showed that HycP contains a functional CBM3 that we classified within the CBM3b subtype according to its amino-acids sequence features. Both rCBM3a and rCBM3b were shown to bind to cellulose and straw as it was previously shown for other CBM3a and b [26]. Determined dissociation constants for rCBM3a with these substrates are consistent with previous data obtained from a recombinant miniCipC protein containing the first three modules of CipC, except for the interaction with PAS cellulose [22]. This difference may be explained by the use of different PAS cellulose preparations in both studies, or by the influence of the surrounding domains present in miniCipC, compared to rCBM3a. We showed that rCBM3b exhibits an overall reduced affinity for crystalline cellulose compared to rCBM3a. A plausible explanation for the difference between both CBM is that in CBM3b a stretch of about 3–4 amino-acids replaces the 4′ β-strand containing a conserved tyrosine in the CBM3a. This latter aromatic amino-acid is involved in one of the important stacking interactions between CBM3a and planar crystalline cellulose [27], [28], [29]. In CBM3b from HycP, no aromatic acid is present in this stretch which might be the cause of its weaker interaction for crystalline cellulose.

**Table 2:** Summary of the compared properties of CipC and HycP.

Property	CipC	HycP
Modules found	CBM3a	CBM3b
	Cohesins	HYR modules
	X2	
Preferred binding substrate	Crystalline celllulose	Amorphous cellulose
Hydrolytic activity	No	Not detected
Role in cell adherence to cellulose	Partial	No
Role in cell growth on cellulose	Yes	Not detected

The presence of the large HycP protein composed of 23 repeats of the HYR module with unknown function, together with a functional CBM3b, raises the question of its function in *C. cellulolyticum*. HYR domains were initially discovered in eukaryotes but are also found in prokaryotes where they are inserted in some surface proteins, associated with some glycoside hydrolase modules (chitinases) or are part of hypothetical proteins [24]. In the bacterial kingdom, proteins containing multiple HYR modules like HycP are mostly found in marine or freshwater environment microorganisms, where their function is again unknown. The only protein predicted to contain a similar domain organization as HycP, *i.e.* many HYR domains associated to a CBM3, is found in *Clostridium sp* BNL1100. This strain is very close to *C. cellulolyticum* and was isolated from corn stover [37]. Both HYR domain containing proteins share 78% sequence identity. The search for other proteins containing HYR module(s) accompanied with a CBM in the NCBI data base resulted in three proteins: a 390-kDa protein from *A. cellulolyticus* CD2 (accession number ZP_09466191.1) which is predicted to be composed of a peptidase_C11 domain in the N-terminal part followed by two HYR modules with a CBM3 at the C-terminus, and two putative xylanases which both contain a CBM4_9 and a Family-10 glycoside hydrolase module (accession number gi|147830786, *Clavibacter michiganensis subsp. michiganensis NCPPB 382*; accession number YP_001360820, *Kineococcus radiotolerans SRS30216*). No HYR module containing protein is found in other described cellulolytic clostridia as *C. thermocellum*, *C. cellulovorans* or *C. papyrosolvens*. HycP is the only HYR modules containing protein in *C. cellullolyticum*. The function of the HYR domains in any of these bacterial proteins is unknown.

We explored the possible role of HycP in the light of the function of CBM3-containing proteins found in many other cellulolytic bacteria. CBM3b is usually associated with a Family-9 glycoside hydrolase module in cellulases [26]. Enzymatic assays performed on mini-HycP did not show any glycoside hydrolase activity towards cellulosic substrates, suggesting that the whole protein HycP is devoid of hydrolytic activity on cellulose. This is consistent with the low sequence similarity of the molecule with any known catalytic module. Another function reported for the CBM3-containing proteins is to sense the substrate as it was described in *C. thermocellum*. Membrane sensor proteins displaying a CBM3 and an anti-sigma factor domain were reported to trigger expression of genes related to the cellulolytic system in the presence of cellulose [38]–[39]. Direct involvement of HycP in the carbohydrate sensing process is however unlikely, since HycP is mainly secreted. In addition, neither the growth on cellulose nor the composition of the cellulosomes are altered when the protein is missing (data not shown). These data strongly suggest that HycP has no direct or indirect role in carbohydrate-sensing. The third function of CBM3-containing proteins is to mediate binding of the whole cell to cellulose. This interaction is established by cellulosomal scaffolding proteins which may contain CBM3a or CBM3b [6], [12], [19], [20]. Our results indicate that HycP is not involved in cell binding to cellulose since no differences were observed in cell adherence to cellulose or growth on cellulose between MTL*hycP* mutant and wild-type strains. Altogether these results suggest that the protein is not essential for cellulose hydrolysis, and its function remains unclear. The gene encoding HycP is only found in *C. cellulolyticum* and the related BNL1100 strain, suggesting a recent evolution of both strains, which may result of an adaptation to their specific environment. Similar to the Fn3 domain, HYR modules belong to the immunoglobuline-like fold [24]. It has been reported that Fn3 domains may modify the cellulose surface helping hydrolysis by the cellulases bearing this module [40]. It is possible that HYR modules displays this property, and the association of 23 HYR modules together with a CBM3b found in HycP may further enhance cellulose surface modification. This putative benefit is not observed when *C. cellulolyticum* is grown on cellulose, but we observed that the secretion of HycP seems to hamper fitness of *C. cellulolyticum* wild-type strain on soluble sugars. Indeed, the generation time of the mutant MTL*hycP* strain grown on cellobiose is reduced by 25% compared to wild-type. The persistence of the gene *hycP* through the evolution of *C. cellulolyticum*, suggests that this protein brings a benefit, putatively through an ancillary function, which may be useful in specific environments encountered by the bacterium and which has yet to be identified.

The putative involvement of CipC in cell adhesion of *Clostridium cellulolyticum* to cellulose was addressed in the present study. The scaffolding cellulosomal protein has been reported to be involved in cell adherence of several cellulosomes-producing bacteria [6], [12], [19], [20]. In C. *thermocellum*, the AD2 mutant failed to attach the cellulosomes at the cell surface and consequently to bind to cellulose [6], [17]. This mutant was found to lack the cellulosomal protuberances observed in the wild-type at the cell surface, highlighting the link between cellulosomal protuberances and adhesion of the cells to cellulose. In contrast in *C. cellulolyticum*, no protuberances were observed at the surface of *C. cellulolyticum* wild-type strain and CipC is only partly involved in cell binding to cellulose. It is worth noting that in other cellulolytic bacteria as *B. cellulosolvens, A. cellulolyticus* or *C. cellulovorans,* protuberances were also observed, and in all these strains, a molecular mechanism is proposed to tether the cellulosomes to the cell surface [12], [18], [19]. Analysis of the *C. cellulolyticum* genome failed to identify genes that encode any putative cellulosome cell surface anchoring proteins homologous to EngE from *C. cellulovorans*, or any predictable cellulosome cell surface anchoring adaptator protein. The lack of cell surface protuberances supports the possibility that, in *C. cellulolyticum*, no specific mechanism is devoted to the anchorage of cellulosomes to the cell surface. Since no protuberances are observed, the part of the adherence found to be due to CipC in our experiments may occur through other mechanisms. The hydrophilic modules (X2) of the scaffolding protein may exhibit some affinity for the peptidoglycan as suggested for *C. cellulovorans* [20]. Another possibility is that during the secretion process of the large CipC protein, the CBM3a module may be transiently exposed at the cell surface, allowing its participation in adherence of the cells to cellulose.

The lack of protuberances and the fact that CipC is only partly involved in the mechanism of adhesion to cellulose, suggests that other mechanisms may participate in cell binding to cellulose. Other mechanisms as bacterial glycocalyx or pili were found to be important for bacterial cell adhesion to cellulose [8]. Filamentous fibrillar appendages are reported to be important factors for adhesive properties of bacteria, biofilm formation and colonization. Two types of pilus are described in gram positives bacteria. The first is covalently linked to the peptidoglycan via the action of a sortase which recognizes a LPXTG motif in the protein [41]–[42]. And the second is the gram-negative-like type IV pilus [43], [44], [45]. It is not known whether *C. cellulolyticum* displays a surface glycocalyx, and no gene encoding sortase and any LPXTG motif containing protein could be found in the *C. cellulolyticum* genome sequence. But the *C. cellulolyticum* genome was reported to encode putative type IV pilus components [46]. As demonstrated in the case of *Ruminococcus albus*, this kind of pilus may also be involved in adhesion of *C. cellulolyticum* to cellulose [43], [44]. Other cell surface proteins containing SLH modules and CBM may also be involved in cell binding to cellulose, as it was suggested in *Caldicellosiruptor saccharolyticus* [47]. In *C. cellulolyticum* it was previously reported that genes encode proteins containing some SLH module(s), together with one or two CBM belonging to families, 9, 17, or 28, reported to bind to cellulosic substrates [21]. Proteins containing these CBMs may therefore participate in the adhesion mechanism(s) of *C. cellulolyticum* to cellulose. The role of type IV pilus, and other SLH and CBM containing proteins, in adherence of *C. cellulolyticum* to cellulose will be investigated in the future.

## Materials and Methods

### Bacterial strains, plasmids, and media

*Escherichia coli* DH5α (Life Technologies), *E. coli* BL21(DE3) (Life Technologies), and *E. coli* SG13009(pREP4) were grown at 37°C in Luria-Bertani medium supplemented with appropriate antibiotics (100 µg.ml^−1^ of ampicillin, 50 µg. ml^−1^ of kanamycin). *C. cellulolyticum* H10 ATCC 35319 [48] and mutants were grown anaerobically at 32°C on basal medium [49] supplemented with either 2 g.L^−1^ cellobiose (Sigma-Aldrich) or 5 g.L^−1^ cellulose, Sigmacell 20, (Sigma-Aldrich) or Avicel microcrystalline cellulose (PH101, Fluka, Buchs, Switzerland). When necessary, thiamphenicol (5 µg.ml^−1^), erythromycin (2.5 µg.ml^−1^), or tetracyclin (5 µg.ml^−1^) were added to the medium. Colonies of recombinant *C. cellulolyticum* strains carrying mutation in their chromosomes were isolated under the anaerobic atmosphere of a glove box (N_2_-H_2_, 95∶5 [vol/vol]), on solid basal medium supplemented with 2 g.L^−1^ of cellobiose, 15 g.L^−1^ of agar, and 2.5 µg of erythromycin, supplemented with tetracycline (5 µg.ml^−1^) when necessary for experiments using the complemented strains. Plates were incubated in anaerobic jars under 2×10^5^ Pa of an N_2_-CO_2_ (80∶20 [vol/vol]) atmosphere.

*Clostridium perfringens* (strain CIP 60.61, Institut Pasteur, France) was anaerobically grown at 37°C in standard TGY medium.

*Clostridium thermocellum* DSM wild-type strain was grown anaerobically at 60°C in previously described medium [6].

Vectors and strains used in this study are reported in Table 3. The expression plasmid pET22b (Novagen) was used for the production in *E. coli* of the recombinant rCBM3b module, the recombinant protein rHyr, corresponding to the three last HYR modules of the HycP, and the recombinant rCBM3a of CipC in *E. coli*. pET28a was used for the production of the mini-HycP in *E. coli*. A derivative of pMTL007 was used for inactivation of *hycP* or *cipC* genes in *C. cellulolyticum*. pSOScipC, pSOS954, pSOSzero-Tc were used for complementation of the *C. cellulolyticum* mutant strain [23], [33], [35].

**Table 3:** Bacterial strains and vectors used in this study.

Strain or plasmid	Relevant characteristics	Source or reference
*E. coli* DH5a	F^−^ *endA1 hsdR17*(rK^−^ mK^+^) *supE44 thi-1 λ gyrA96 relA1Δ*(*lacZYA argF*) *U169* (Φ80 *lacZ Δ*M15) *recA*	Roche Diagnostics
*E. coli* SG13009(pREP4)	F^−^ *his pyrD Δlon-100 rpsL* (pREP4)	Qiagen
*E. coli* BL21(DE3)	F^−^ *ompT hsdS* (rB^−^ mB^−^) *gal dcm* (DE3)	Novagen
*C. cellulolyticum* H10	Wild-type, ATCC35519 DSM 5812	DSMZ
*C. cellulolyticum* MTL*hycP*	ATCC35319, *hycP*: intron, Erm^r^ containing the vector pMTL007*hycP*	This study
*C. cellulolyticum* MTL*cipC*	ATCC35319 derivative, *cipC*: intron, Erm^r^	This study
*C. thermocellum*	Wild-type DSM	DSM [6]
*C. perfringens*	Strain CIP 60.61	Institut Pasteur
pET22b+	*E. coli* expression vector; Ap^r^	Novagen
pET28a	*E. coli* expression vector; Kan^r^	Novagen
pETHyr	pET22b+ derivative carrying the735-bp NdeI-XhoI fragment encoding the last three HYR modules of HycP	This study
pETCBM3b	pET22b+ derivative carrying the 468-bp NdeI-XhoI fragment encoding the CBM3b module of HycP	This study
pETCBM3a	pET22b+ derivative carrying the NdeI-XhoI fragment encoding the CBM3a module of CipC	This study
pETMiniHycP	pET28a derivative carrying the 2135-bp NcoI-XhoI fragment encoding the MiniHycP	This study
pMTL007	*E. coli/Clostridium* shuttle vector (ColE1, pCB102)Ll.*ltr*Bintron (*erm*BtdRAM2) under the control of P*fac*, *ltr*A; Cm^r^/Tm^r^	[30]
pMTL007*cipC*	pMTL007 derivative targeting *cipC* (locus Ccel_0728)	This study
pMTL007*hycP*	pMTL007 derivative targeting *hycP* (locus Ccel_1491)	This study
pSOSzero-Tc	*E. coli/Clostridium* shuttle vector (ColE1, pIM13); Ap^r^,Tc^r^	[33]
pSOS954	*E. coli/Clostridium* shuttle vector (ColE1, pIM13); P*thl* carrying a -35 mutated box expression cassette from *C. acetobutylicum*, Ap^r^,Erm^r^	[35]
pSOS955	pSOSzero-Tc derivative carrying SalI-SalI expression cassette from *C. acetobutylicum* from pSOS954, Ap^r^,Tc^r^	This study
pSOS*cipC*	*E. coli/Clostridium* shuttle vector (ColE1, pIM13) carrying *cipC* gene under the control of the mutated P*thl*, Ap^r^,Erm^r^	[31]
pSOS955*cipC*	pSOS955 derivative carrying 4677-bp BamHI-SwaI fragment from pSOS*cipC*, Ap^r^,Tc^r^	This study

Ap^r^, ampicilline resistance; Erm^r^, erythromycin resistance; Kan^r^, kanamycine resistance; Cm^r^/Tm^r^, chloramphenicol/thiamphenicol resistance; Tc^r^, tetracycline resistance.

### Growth measurements

Growth on cellobiose-supplemented basal medium was followed by monitoring optical density at 450 nm over time. When cultured on 5 g.L^−1^ Sigmacell, growth measurements were based on protein content measurement as described previously [49].

### Construction of *cipC* and *hycP* mutations in *Clostridium cellulolyticum*

Gene inactivation in *C. cellulolyticum* was performed using the ClosTron technology as described by Heap *et al.*, 2007 with minor modifications [30]. The integration sites in the target genes and the primers used to retarget the Ll.LtrB intron in the pMTL007 (IBS, EBS1d and EBS2, see data S2) were generated by the free Perutka algorithm implemented at http://ClosTron.com. Antisens intron integrations were chosen at position 116|117 for *cipC* and 829|830 for *hycP* downstream of the start codon. Specific *cipC* and *hycP* target primers IBS, EBS1d and EBS2 and the universal primer EBS universal were used to produce a fragment by overlapping PCR using pMTL007 as the matrix. The fragments were subsequently digested by BsrGI and HindIII and cloned in pMTL007 similarly digested. The retargeted resulting vectors were called pMTL007*cipC* and pMTL007*hycp*.

The vectors were methylated *in vitro* with MspI prior to be transferred in *C. cellulolyticum* by electro-transformation as previously described [50], [51]. The transformed cells were selected using thiamphenicol. Induction of the intron integration was performed by incubation of cells with 3 mM IPTG, and the mutated clones were selected using erythromycin. Clones mutated in *cipC* and *hycP* genes were called MTL*cipC* and MTL*hycP*, respectively.

### Complementation of MTL*cipC* mutant

For MTL*cipC* complementation we used the *cipC* gene previously cloned in an erythromycin resistant pSOS*cipC* vector [31]. As the MTL*cipC* mutated strain already contains erythromycin resistance brought by the mutation in the genome, we used the tetracycline resistant vector pSOSzero-Tc previously constructed [33]. This vector was digested using SalI and ligated with the expression cassette obtained from pSOS954 digested by the same enzymes [35]. The resulting *E. coli*-*C. cellulolyticum* shuttle expression vector called pSOS955 was then digested with BamHI and EheI, and ligated with the *cipC* gene excised from pSOS*cipC* using BamHI and SwaI. The strain SG13009 (pREP4) was used as the recipient strain for transformation. The resulting vector was called pSOS955*cipC*. The vectors pSOS955*cipC* and pSOSzeroTc were transferred in MTL*cipC* strain thereby generating the *cipC* complemented strain MTL*cipC* (pSOS955*cipC*) and the control strain MTL*cipC* (pSOSzero-Tc), respectively.

### Cloning of the genes encoding rCBM3a, rCBM3b, rHyr and mini-HycP in *E. coli*

All primers used in this study are presented in data S2. rCBM3a is designed to fuse the CBM3a from *cipC* (from amino-acid 27 to 187) in frame with a sequence of 6 histidine residues at its C-terminus. The pET-CBM3a was obtained by PCR on the genomic DNA of *C. cellulolyticum* using the forward CBM3aNdef and reverse CBM3aXhoIR primers, respectively. The amplicon was subsequently digested with NdeI and XhoI and cloned in a NdeI-XhoI linearized pET22b(+) thereby generating pET-CBM3a.

The 468 bp region of the *hycP* that encodes the CBM3 (from amino-acid 2187 to 2343) was amplified by PCR using the primers CBMHyCPNdeD and CBMHyCPXhoR whereas the 760 bp region of the gene *hycP* that encodes the three last HYR modules (from the amino-acid 1950 to 2192) was amplified by PCR using the oligonucleotides HyrNdeD and HyrXhoR. These primers introduced NdeI and XhoI sites upstream and downstream of the coding sequence, respectively. One ATG initiation codon was present in the forward primer. Amplicons were digested by NdeI and XhoI, and cloned in a similarly digested pET22b(+) vector. The resulting vectors pET-CBM3b and pET-Hyr contained the coding sequence for the rCBM3b and rHyr proteins fused in frame with a sequence encoding six histidine residues at their C-terminus, respectively.

Mini-HycP was designed to fuse the region starting from the amino-acid 58 to 609 to the region 2190 to 2343, in frame with a sequence encoding six histidine residues at its C- terminus. The gene encoding the Mini-HycP was generated by overlapping PCR performed on genomic DNA from *C. cellulolyticum*: the first PCR generated a 1681bp fragment using 1491_175NcoID and 1491_1830HyrR primers and the second one generated a 492bp-fragment using 1491_6559CBMD and CBMHyCPXhoR primer. The final amplicon was generated by mixing the two overlapping PCR fragments, and extended using primers 1491_175NcoID and CBMHyCPXhoR. The final amplicon was digested with NcoI and XhoI and cloned in a NcoI – XhoI linearized pET28a thereby generating the pET-miniHycP.

Plasmid pET-CBM3b, pET-Hyr, pET-miniHycP, pET-CBM3a were used to transform the BL21(DE3) strain to produce the corresponding recombinant proteins.

### Production and purification of the recombinant proteins

Recombinant *E. coli* BL21(DE3) were grown at 37°C with shaking to an optical density at 600 nm of 1.0, Isopropyl-β-D-thiogalactopyranoside (IPTG) was added to a final concentration of 200 µM, and the cultures were incubated overnight under shaking at 25°C except for BL21(DE3)(pET-mini-HycP) strain for which induction of the heterologous gene expression was performed at 20°C. The cells were then harvested by centrifugation for 15 min at 6000 *g* and broken in a French press. After centrifugation of the crude extract (10 min, 4°C, 10000 *g*) the His-tagged proteins present in the supernatant were loaded on a column of Ni-nitrilotriacetic acid superflow resin (Qiagen, Hilden, Germany) equilibrated with 20 mM Tris-HCl (pH 8), and eluted using the same buffer supplemented with 60 mM imidazole. After concentration by ultrafiltration (Vivaspin 20, 10 kDa cutoff, Sartorius, Germany), the proteins were further purified by an anion exchange chromatography (Hi-trap Q-sepharose, GE Healthcare, Buckinghamshire, UK). The fractions of interest were pooled, dialyzed, and concentrated in 20 mM Tris-HCl (pH 8) by ultrafiltration (Vivaspin 20, 10 kDa cutoff, Sartorius, Germany). The absorbance at 280 nm was measured and the protein concentration was determined using their specific extinction coefficient. The purified recombinant rHyr protein was sent to Eurogentec France for polyclonal antibody production using the speedy 28-days protocol.

### PAGE and Western blot analysis

Sodium dodecyl sulfate-polyacrylamide gel electrophoresis (SDS-PAGE) was performed using a vertical electrophoresis system. Gels were stained with Coomassie blue or were electroblotted onto nitrocellulose membranes (Hybond-ECL, GE Healthcare, Buckinghamshire, UK). Membranes were probed with polyclonal rabbit antibodies raised against rHyr protein, or CipC [25]. Primary antibodies were detected using anti-rabbit horseradish peroxidase conjugate (Promega, Madison, WI) and a chemiluminescent substrate (Millipore, Billerica, MA). When necessary, samples were precipitated by 10% ice-cold TCA (v/v), and the pellet was washed twice with acetone, dried and solubilized in loading SDS-PAGE buffer.

### Protein binding assays

Binding of protein to polysaccharides were examined by incubating 40 µg of protein with 10 mg of Avicel microcrystalline cellulose (PH101, Fluka, Buchs, Switzerland), or hatched straw (Valagro, Poitiers, France) in 20 mM phosphate buffer (pH 7.0) in a 250 µl final volume during 1 hour at 4°C under gentle shaking. After centrifugation the pellet was washed twice with the same buffer and a sample of the pellet fraction (bound proteins) and of the supernatant (unbound proteins) were analyzed by SDS-PAGE.

Binding constants were determined as formerly described [22]. Binding constants were determined for rCBMs incubated with Avicel PH101, Sigmacell 20 (Sigma), Phosphoric Acid Swollen cellulose (PASC), bacterial microcrystalline cellulose (BMCC) or hatched straw (Valagro, Poitiers, France). PASC was obtained from Avicel PH101 as previously described [52], BMCC and hatched straw were obtained as previously described [53], [54] respectively.

### Cell adherence assay

Binding assays protocol was based on the previously described protocol with modifications [55]. In a glovebox, *C. cellulolyticum* cells at exponential growth phase were mixed with rich medium buffer to reach an optical density of 0.5 at 450 nm. A volume of 2 mL of cell suspension was transferred in 15 mL Hungate tubes with a strip of filter paper, or nitrocellulose (80×10 mm), saturated or not 1 hour at room temperature with 4% BSA. Tubes were then incubated 1 hour with gentle agitation and optical density at 450 nm from supernatant was measured. Adhesion percentage was deduced from optical density measurement of an assay compared with a control where no filter paper or nitrocellulose was added. The reported values presented are the mean of 3 triplicates performed in at least 3 independent experiments.

### Scanning electron microscopy

SEM experiments were performed on filter paper after 3 days of growth with *C. cellulolyticum* or 1 day with *C. thermocellum*. A piece of the filter paper was incubated with 2.5% glutaraldhehyde in PBS buffer for 30 minutes. Samples are then washed in distilled water and incubated with osmium tetroxyde (4%) for 20 minutes, washed and then gently incubated with 5 ethanol baths containing increasing concentration of ethanol, from 50% to 100%, for 10 minutes each. Filter paper was then incubated two minutes with a 50∶50 [vol/vol] solution of ethanol and hexamethyldisilazane (HMDS) and then 100% HMDS until complete evaporation, and kept dried for gold/palladium alloy coating. Samples were observed in the next few hours using a scanning electron microscope JSM 6320F (Jeol), at the CINaM microscopy service (Centre Interdisciplinaire de Nanosciences de Marseille, CNRS, Marseille).

### Protein sequence analysis

Amino acid sequences were compared with those in the NCBI database using the BLAST program (http://blast.ncbi.nlm.nih.gov.gate1.inist.fr/Blast.cgi) [56]. Predictions of domains from amino acids sequences were performed using the Simple Modular Architecture Tool (SMART) (http://smart.embl-heidelberg.de/) [57] and the PFAM protein families database (http://pfam.sanger. ac.uk) [58]. Prediction of signal peptide cleavage sites and transmembrane segments was performed using SIGNALP V4.0 program (http://www. cbs.dtu.dk/services/SignalP) [59] PREDIction of Signal peptide tool (http://www.predisi.de/index.html) [60] and TMHMM 2.0 program (http://www.cbs.dtu.dk/services/TMHMM/) [61]. Multiple sequence alignments were performed with the ClustalW2 program (http://www.ebi.ac.uk/Tools/msa/clustalw2/) [62].

## Supporting Information

Data S1 **Amino-acid sequence alignment of HYR modules identified in HycP.** Sequences alignment has been performed using ClustalW2. Stars and grey box indicate identical residues; double dot, strongly similar residues; simple dot, weakly similar residues. Sequence of HYR modules were delimited and numbered as shown in figure 1A.
(TIF)

Data S2 **Primer sequences used in the present study.**
(TIF)
