# Supplementary figures and images for: Are Cellulosome Scaffolding Protein CipC and CBM3-Containing Protein HycP, Involved in Adherence of Clostridium cellulolyticum to Cellulose?
Source: PLoS One. 2013 Jul 25;8(7):e69360. doi: 10.1371/journal.pone.0069360 (PMC3723904; doi:10.1371/journal.pone.0069360)

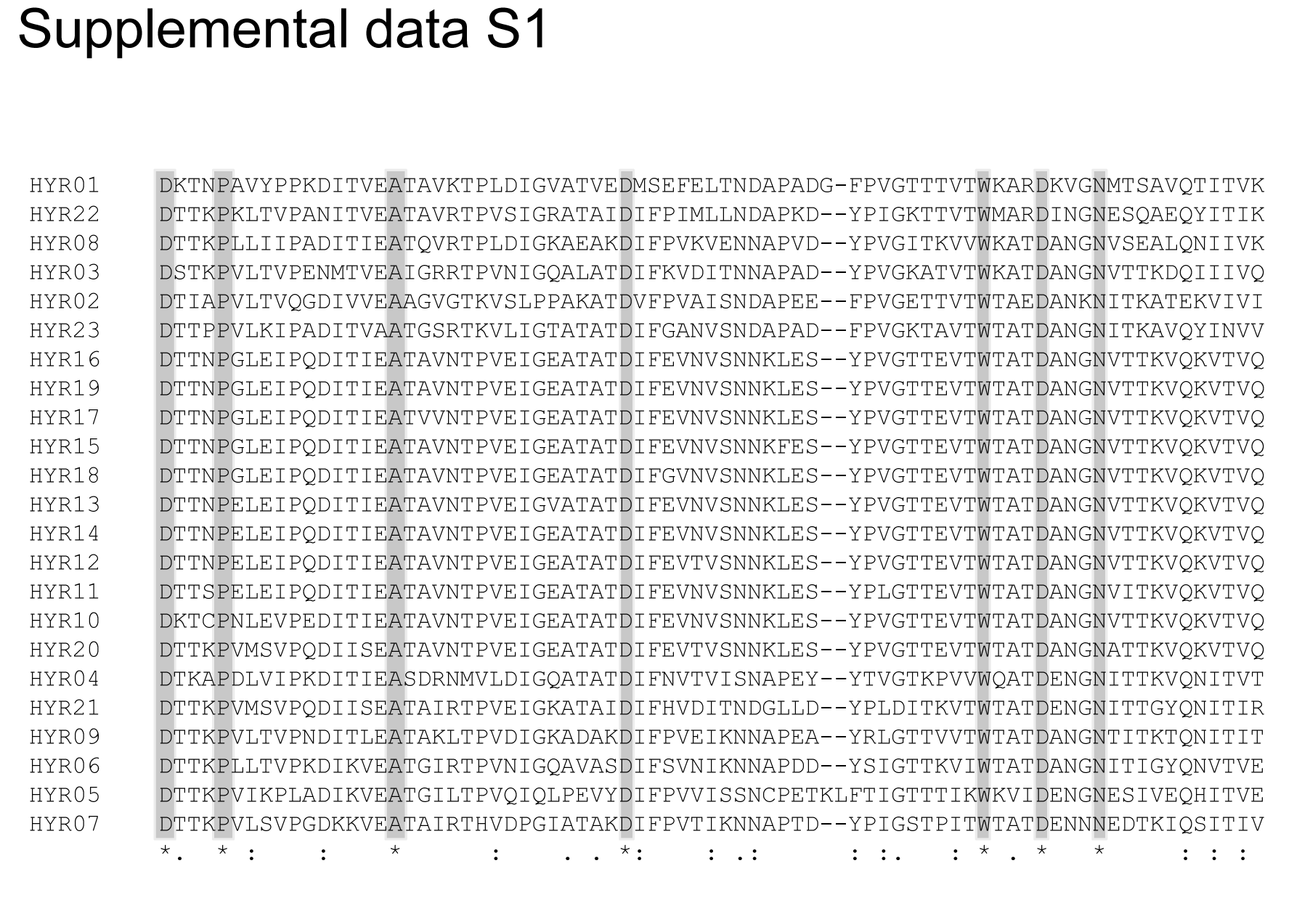

Supplement: Data S1 — Amino-acid sequence alignment of HYR modules identified in HycP. Sequences alignment has been performed using ClustalW2. Stars and grey box indicate identical residues; double dot, strongly similar residues; simple dot, weakly similar residues. Sequence of HYR modules were delimited and numbered as shown in figure 1A. (TIF) [file pone.0069360.s001.tif]

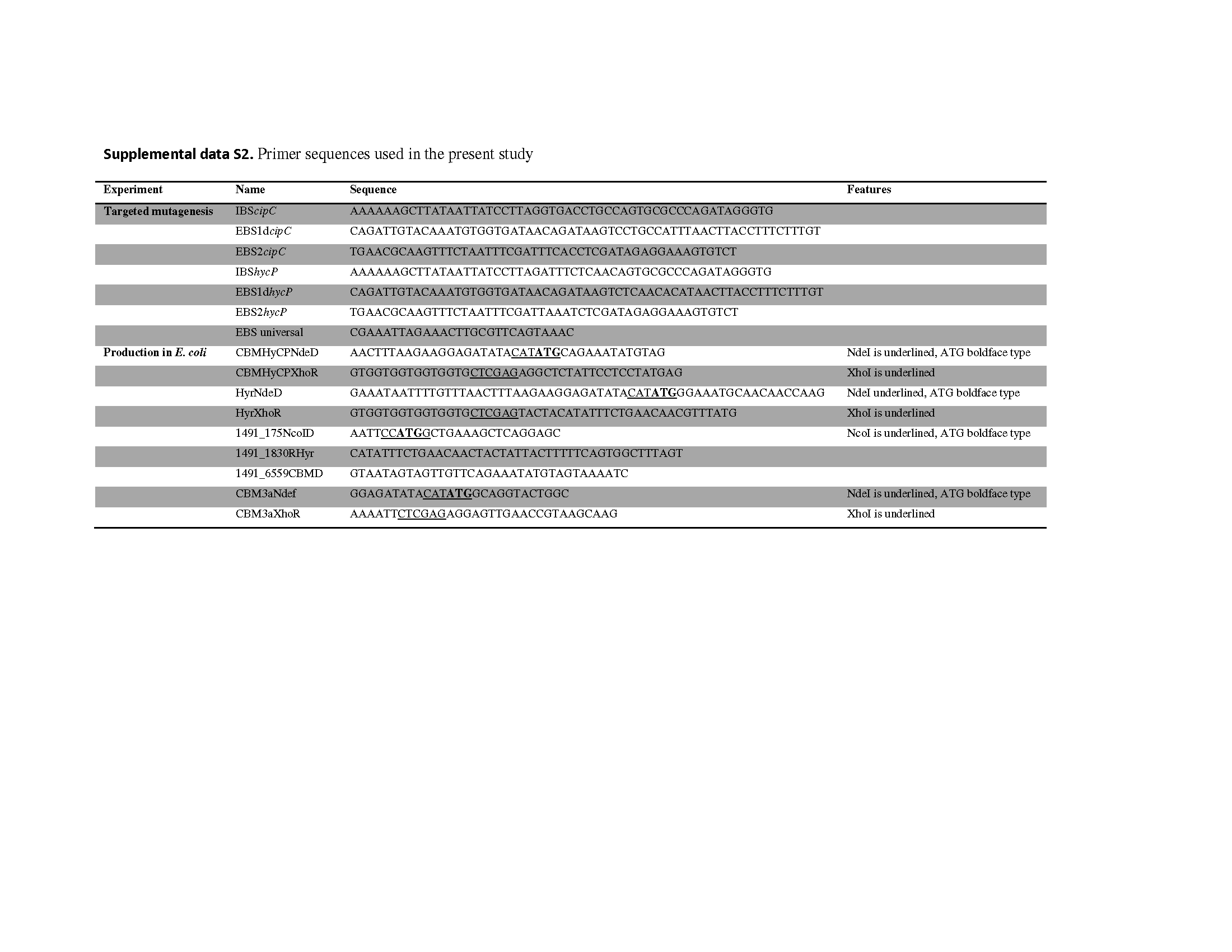

Supplement: Data S2 — Primer sequences used in the present study. (TIF) [file pone.0069360.s002.tif]
